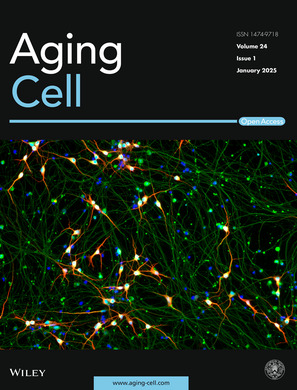# Additional Cover

**DOI:** 10.1111/acel.14479

**Published:** 2025-01-08

**Authors:** Todd W. Dowrey, Samuel F. Cranston, Nicholas Skvir, Yvonne Lok, Brian Gould, Bradley Petrowitz, Daniel Villar, Jidong Shan, Marianne James, Mark Dodge, Anna C. Belkina, Richard M. Giadone, Sofiya Milman, Paola Sebastiani, Thomas T. Perls, Stacy L. Andersen, George J. Murphy

## Abstract

Cover legend: The cover image is based on the article *A longevity‐specific bank of induced pluripotent stem cells from centenarians and their offspring* by George Murphy et al., https://doi.org/10.1111/acel.14351.